# Computational screening and molecular dynamics reveal curcumin III and taxifolin as potential thyroid receptor modulators for hypothyroidism therapy

**DOI:** 10.3389/fendo.2026.1727415

**Published:** 2026-02-16

**Authors:** Babatunji Emmanuel Oyinloye, Adetola Ibukunoluwa Adewale, Shalom Oluwafunke Adeyemi, Omotola Martha Fajana, Olutosin Samuel Olusola, Oluwatoyin Mary Oyinloye, Aderonke Moyosola Ayeni, Ayodeji Benjamin Akawa, Olajumoke Tolulope Idowu, Austine Idowu Ibikunle, Folake Olayinka Olojo, Basiru Olaitan Ajiboye

**Affiliations:** 1 Institute for Drug Research and Development, S.E. Bogoro Center, Afe Babalola University, Ado-Ekiti, Nigeria; 2 Phytomedicine, Biochemical Toxicology and Biotechnology Research Laboratories, Department of Biochemistry, College of Sciences, Afe Babalola University, Ado-Ekiti, Nigeria; 3 Biotechnology and Structural Biology (BSB) Group, Department of Biochemistry and Microbiology, University of Zululand, KwaDlangezwa, South Africa; 4 Department of Biology, Wake Forest University, Winston Salem, NC, Nigeria; 5 Department of Biological Sciences, College of Sciences, Afe Babalola University, Ado-Ekiti, Nigeria; 6 Medical Biochemistry Unit, College of Medicine and Health Sciences, Afe Babalola University, Ado-Ekiti, Nigeria; 7 Industrial Chemistry Unit, Department of Chemical Sciences, College of Sciences, Afe Babalola University, Ado-Ekiti, Nigeria; 8 Department of Community Medicine, Federal Teaching Hospital (FETHI), Ido-Ekiti, Nigeria; 9 Department of Community Medicine, College of Medicine and Health Sciences, Afe Babalola University, Ado-Ekiti, Nigeria; 10 Department of Chemical Sciences, Dominion University, Ibadan, Nigeria; 11 Phytomedicine and Molecular Toxicology Research Laboratory, Department of Biochemistry, Federal University Oye-Ekiti, Oye-Ekiti, Nigeria

**Keywords:** hypothyroidism, molecular docking, molecular dynamics simulation, phytochemicals, curcuma longa, moringa oleifera, nigella sativa, thyroid receptors

## Abstract

Hypothyroidism is a condition marked by inadequate thyroid hormone production. It is typically treated with Levothyroxine, which, despite its effectiveness, can cause adverse effects on metabolism and the cardiovascular system. In this study, an in silico approach was used to screen phytochemicals from *Curcuma longa*, *Moringa oleifera*, and *Nigella sativa* for their potential to activate the thyroid receptor. A total of 439 compounds were docked against Thyroid Receptor Beta 1 (TRβ1) and Thyrotropin-Releasing Hormone Receptor (TRHR) using AutoDock Vina. Among them, valoneic acid dilactone, curcumin III, taxifolin, luteolin, lophenol, and stigmastanol were identified from the three plants as performing better than others. These compounds were further evaluated with ADMET predictions and molecular dynamics simulations to assess their drug-like properties and stability. Lophenol from *Nigella sativa* showed the highest binding affinity to TRβ1 (-13.62 kcal/mol) with an inhibition constant of 0.1037 nM, surpassing levothyroxine (-11.60 kcal/mol and 6.63 nM). Also, stigmastanol from *Nigella sativa* showed the highest binding affinity to TRHR (-8.71 kcal/mol) with an inhibition constant of 415 nM, surpassing levothyroxine (-7.64 kcal/mol and 2530 nM). However, the two compounds exhibited some unfavourable ADMET properties that could be improved through formulation science. Curcumin III and taxifolin demonstrated favourable ADMET properties, including optimal solubility, lipophilicity, and polar surface area, suggesting good oral bioavailability. Molecular dynamics simulations were carried out using Desmond. The results revealed that curcumin III, lophenol, and taxifolin maintained strong stability, with RMSD values of 1.70 ± 0.005 Å, 2.17 ± 0.012 Å, and 2.50 ± 0.014 Å, respectively, for TRβ1, and RMSD values of 3.82 ± 0.014 Å, 4.29 ± 0.019 Å, and 3.31 ± 0.017 Å, respectively, for TRHR. This study identified Curcumin III from *Curcuma longa* and taxifolin from *Moringa oleifera* as promising natural compounds for the treatment of hypothyroidism. Further validation through *in vitro*, *in vivo*, *and ex vivo* studies is recommended.

## Introduction

Hormones are chemical substances produced by endocrine glands and enter the bloodstream to control several bodily functions. These endocrine glands include the pituitary, adrenal, gonad, thyroid, parathyroid, and pancreatic glands (1). Scientific research has shown that the primary endocrine gland to develop during embryonic development is the thyroid gland (2, 3). Thus, it is regarded as a basic endocrine gland (4). The inability of the thyroid gland to produce adequate thyroid hormones - thyroxine (T4) and triiodothyronine (T3) (which are essential for the metabolic needs of the body) results in the disease condition known as hypothyroidism or under-active thyroid (5). This is brought about by several factors, namely, (a) insufficient stimulation of the thyroid gland by the hypothalamus/pituitary gland, (b) inability of the thyroid gland to receive proper messages from the brain to produce more hormones, (c) thyroid surgery or exposure to radiation (6). Hypothyroidism is a widespread condition with the capacity to culminate in destructive health consequences that affect humans globally (7). This condition alters nearly the entire body system, and it can present as asymptomatic or symptomatic (5). More so, it could be transient or permanent (8). The manifestations and indicators indicative of thyroid impairment are characterized by their nonspecific and non-diagnostic nature, particularly in the initial stages of disease manifestation. (9). If hypothyroidism is detected in its early stages, it is easy to treat, but if proper treatment is neglected, it can trigger life-threatening complications across many organ systems, where the circulatory system is the most strongly investigated target (10). These complications include infertility, hypertension, neuromuscular dysfunction, dyslipidemia, and cognitive impairment (6). The occurrence of hypothyroidism increases with age, particularly in people above 50 years, with a higher occurrence in women than in men. (11) It has been assessed that about 5% of the general population of humans are affected by hypothyroidism, while a further estimation of 5% of the human population is not identified through diagnosis (12).

There are different types of hypothyroidism, including primary, secondary, tertiary, and peripheral types. Primary hypothyroidism is a product of a deficiency of thyroxine, while the deficiency of thyroid-stimulating hormone (TSH) results in secondary hypothyroidism. Peripheral hypothyroidism occurs due to a shortage of thyrotropin-releasing hormone (5). Both the secondary and tertiary hypothyroidism make up central hypothyroidism. Both the central hypothyroidism and peripheral hypothyroidism (extrathyroidal) are uncommon, with only about less than 1% cases (5). In days gone by, a deficiency of iodine was the common cause of hypothyroidism because iodine mainly comes from diet, and the body does not make iodine (13). Nowadays, the major origin of primary hypothyroidism is Hashimoto’s disease, an autoimmune disorder in which the body produces antibodies that harm thyroid tissues, causing the thyroid gland to deteriorate gradually and suppress the production of the thyroid hormone (14). More than 99% of affected patients are diagnosed with primary hypothyroidism (12). Other causes of underactive thyroid include stress, some medications and hormonal fluctuations owing to pregnancy and menopause Signs (15) and symptoms of underactive thyroid include fatigue, depression and apathy, weight gain, constipation, insomnia, hair loss, infertility, anxiety, panic attack, tingling hands and feet, hoarse voice, lowered immunity, cold intolerance, lethargy, low libido, headache, slow healing and so on (8, 9).

Majority of individuals with hypothyroidism will require protracted thyroid hormone therapy (12). Levothyroxine, the recommended drug for the treatment of hypothyroidism, unfortunately has unfriendly backsides, though it normalises the quantity of the thyroid-stimulating hormone (TSH) (6). Excessive or under-treatment with levothyroxine usually has negative side effects on glucose and lipid metabolism and cardiovascular function. It decreases physical capability owing to impaired cardiac output, causing muscle weakness and reducing the proper functioning of the lungs, hampering good growth and development, endangering reproductive function, cognitive function, bone metabolism, and gastrointestinal function (16). And for that reason, there is an accelerating interest in alternative treatments such as the use of bioactive compounds in medicinal plants. These would have the potential to support thyroid function, normalize thyroid hormones and inhibit side effects. Certain bioactive compounds may support patients on thyroid hormone replacement therapy by improving their overall health and reducing the need for higher doses of medication.

This study aims to utilize computational tools to investigate the potential therapeutic effects of bioactive compounds from these three medicinal plants - *Moringa oleifera*, *Curcuma longa*, and *Nigella sativa* that can be used as alternatives to the conventional drug (levothyroxine) with minimal or zero side effects, having the potential to target the Thyroid receptor beta 1 and Thyrotropin-Releasing Hormone receptor.

*Moringa oleifera* Lam. belonging to the Moringaceae family, sometimes called the ‘miracle tree’ as every part of the plant has nutritional and pharmacological benefits (17, 18). Previous reports showed its medicinal effects in hypertension, diabetes, inflammatory conditions, cancer, osteoporosis and thyroid dysfunctions (19–22). *M.* oleifera, especially its leaf extract has been shown to regulate thyroid hormones in different animal studies (23; –20, 24). Curcuma *longa* (F. Zingiberaceae), commonly called turmeric, is a rhizomatous plant with origin in India and cultivated widely in China, West Africa, East Africa and some other tropical areas (25). Tumeric is used in Ayurvedic medicine (India) and Chinese Traditional Medicine (China) for the treatment of a wide range of illness including cough, cancer, diabetes, ulcers inflammation. The dry rhizome is also used as flavouring agent, having a characteristic yellow colour due to the phenolic compounds ‘curcuminoids’ present (25, 26). *Nigella sativa* (F. Ranunculaceae), an annual herb plant found to grow in different parts of the world though native to Asia, has been referred to as an important medicinal plant. It is commonly referred to as Black seed, and long been used to relieve different ailments as used in Unani, African, Eastern, Arabic, Chinese and Ayurvedic medicine (27). *N. sativa* parts seeds, oils, and extracts have been reported in studies for treatment of asthma, inflammatory disorders, cancer, arthritis, diabetes, conditions resultant from oxidative stress e.t.c (27–29). Also, a number of animal studies have been conducted to evaluate its potential as therapeutics in hormonal dysfunction including hypothyroidism (30–36).

Furthermore, and more specifically, this study is aimed at identifying bioactive compounds that have therapeutic effects on dual-targets (TRβ1 and TRHR), because there are no medicinal plants previously identified in scientific literatures that specifically target both TRβ1 and TRHR simultaneously for hypothyroidism treatment (37). In addition, no medicinal plants have been reported to specifically target TRHR for hypothyroidism (38), thus alluding to the novelty of this study. Moreso, research into taxifolin's role in hypothyroidism is limited (39) and reported findings on curcumin are primarily based on animal studies, rather than computational. Based on available data, very few natural products with a dual-target modulatory effect have been reported for hypothyroidism therapy (40, 41). This is the insight that this study provides computational empirical evidence for, thus providing a compass for further *in vivo* or *in vitro* studies.

## Materials and methods

### Phytochemical library Generation and Ligand preparation

A library of phytochemicals of *Curcuma longa* (158)*, Moringa oleifera* (106), and *Nigella sativa* (175), previously reported in published articles, was obtained from PubChem (https://pubchem.ncbi.nlm.nih.gov/) and IMPPAT online databases (42, 43) in two-dimensional structure (2D) SDF format. The phytochemicals were converted to pdbqt formats using the Open Babel LigPrep command lines in AutoDock Vina (44). The ligands were protonated at physiological pH (~7.4) using Open Babel. Gasteiger–Marsili partial charges, which were well-matched with the AutoDock Vina scoring function were assigned to the ligands.

### Receptor selection, retrieval and preparation

Two proteins involved in hypothyroidism were selected for this study. Three-dimensional (3D) Experimental structures of Thyroid receptor beta 1 (PDB ID: 1NAX) (45) and Thyrotropin-Releasing Hormone receptor (PDB ID: 7X1T) (46) were retrieved from the Protein Data Bank (https://www.rcsb.org) and visualised using PyMOL molecular graphics system (version 2.0, Schrödinger, LLC., New York, NY, USA). Polar hydrogen was added while water molecules, other atoms and the co-crystallised ligands were removed. The proteins were protonated at physiological pH (~7.4) using Open Babel and gasteiger–Marsili partial charges, compatible with the AutoDock Vina scoring function were assigned to the receptors before the molecular docking (44).

### Binding site definition and receptor grid generation

The coordinates of active sites on the receptors (receptor grid), where ligand interaction occurs, were defined by using the co-crystallised ligands to specify the dimensions on Autodock (47). The user-specified grid map point was set to 60 X 60 X 60. The XYZ grid coordinates are 4.01 Å, 21.1 Å, 31.86 Å for 1NAX and 98.97Å, 117.55 Å, and 76.52 Å for 7X1T, respectively.

### Molecular docking

The study employed the PyMOL/Autodock Plugin tool for docking of the phytochemicals against the proteins (47, 48). The co-crystallised ligands were extracted from each receptor on PyMOL and redocked on Audock Vina, while the other ligands were docked using script where the receptor (protein target) was treated as static (rigid) while the ligands’ rotatable bonds were set to be free (flexible). The exhaustiveness was set at 8, and one pose with the lowest RMSD was generated as output. The 3D protein-ligand interaction was generated using the Discovery Studio Visualizer version 2024. The online tool, PLIP, was also used to determine the interacting amino acid residues (49). The ligands from each plant were ordered based on binding affinity and inhibition constant values. The selected ligands were subjected to molecular dynamics simulation.

### Drug likeness and in silico pharmacokinetic profile

Selected pharmacokinetics (ADMET) and drug-likeness properties were conducted using the canonical smile notations of selected lead compounds in ADMET Lab 3.0 (50). These in silico pharmacokinetic and drug-likeness properties included hydrogen bond donor/acceptor, molecular weight, Lipinski’s rule of five, Blood-brain barrier penetration, partition coefficient, etc.

### Molecular dynamics simulation and trajectory analysis

Molecular dynamics (MD) simulation and Trajectory analysis were carried out to validate the docking results obtained earlier and evaluate the stability of lead complexes using the previously described method reported in (51). The docked complexes of lead compounds, from each plant, were subjected to MD simulation alongside the standard drug, levothyroxine, and co-crystallized ligands on each receptor. The system was prepared using the TIP3P solvent model of the System builder module of the Maestro Schrödinger suite (52) while each complex was bound in an orthorhombic box with 10 X 10 X 10 Å dimensions, minimized, overall charges neutralized by adding sodium and chloride ions, and the salt concentration was set to 0.15M, mimicking a physiologic state. Thereafter, each system was allowed to undergo a 200ns simulation in an NPT ensemble at 300K and 1 atm using the OPLS3e force field, following the standard protocols of Desmond simulation package of Schrödinger LLC (53). The data outputs of the simulations, including root mean square deviation (RMSD) and radius of gyration (rGyr) were plotted using Origin version 6.0 (54) while statistical analyses were done using GraphPad Prism version 9.0.0 for Windows, GraphPad Software, San Diego, CA, USA (www.graphpad.com, accessed on 1st March 2025).

### Binding free energy calculations

The binding free energy was calculated using the Molecular Mechanics/Generalized Born Surface Area (MM/GBSA) approach for the molecular dynamic simulation trajectory analysis. (55, 56) This was done using the Prime module in the Maestro environment. In the MM/GBSA methods, the free binding free energy is calculated thus:

(1) $$G=\;E_{bind}+\;E_{el}+E_{vdw}+G_{pol}+G_{np}-TS$$

(2) $$\Delta G_{bind}=\Delta G_{complex}–\Delta G_{protein}–\Delta G_{ligand\;\;}$$

(3) $$\Delta G_{bind}=\Delta H–T\Delta S$$

(4) $$\Delta G_{bind}=\Delta_{gas}+\Delta G_{solv}–T\Delta S$$

(5) $$\Delta E_{gas}=\Delta E_{int}+\Delta E_{vdw}+\Delta E_{ele}$$

(6) $$\Delta G_{sol}=\Delta G_{GB}+\Delta G_{SA}$$

(7) $$G_{SA}{=}_{\gamma }SASA$$

Where the first three terms in Equation 1 represent energy from bonded, electrostatic and van der Waals interactions respectively while *G_pol,_*and *G_np_* represents polar and non-polar contributions to solvation free energies. *ΔE_gas_, ΔG_solv_*, and *– T*Δ*S* denotes changes in gas phase energy, free solvation energy and total entropy upon ligand binding to the receptor as T is temperature of the systems. *ΔE_gas_*represents change in gas phase internal energy which includes changes in internal energies *ΔE_int_*(bond, angle and dihedral energies), van der Waals energy *ΔE_vdw_* and electrostatic (Coulomb) energies *ΔE_ele_*Meanwhile *ΔG_sol_*is calculated the summation of the polar energy contribution: electrostatic solvation energy *ΔG_GB_*and nonpolar solvation energy contribution *ΔG_SA_* usually estimated using the solvent-accessible surface area (SASA) between the solute and the solvent (57–59).

The plant materials used in this study were *Curcuma longa, Moringa oleifera*, and *Nigella sativa*, while the methods engaged in the study involved the generation and preparation of proteins and ligands, generation of the grid site, molecular docking of the prepared ligands into the active sites, pharmacokinetics analysis of the hit compounds, molecular dynamics simulation of the protein-ligand complex of the hit compounds and the binding energy calculations from the MD trajectories of the hit compounds.

## Result

### Binding affinities and inhibition constant

In this study, the therapeutic of phytochemicals from *Curcuma longa, Moringa oleifera*, and *Nigella sativa* as agonists at the binding sites of Thyroid receptor beta 1 (PDB ID: 1NAX) and Thyrotropin-Releasing Hormone receptor (PDB ID: 7X1T) were assessed. On 1NAX, lophenol (T5) showed the highest binding affinity of -13.62 kcal/mol, while taxifolin is the least performing hit on the receptor with a docking score of -8.28 kcal/mol. The binding affinity of valoneic acid dilactone (T1) is -10 kcal/mol, whereas the co-crystallized ligand, IH5 {3, 5-dichloro-4-[4-hydroxy-3-(propan-2-yl)phenoxy]phenyl}acetic acid) and standard drug, levothyroxine, are -11.44 and -11.60 kcal/mol.

**Figure 1:**
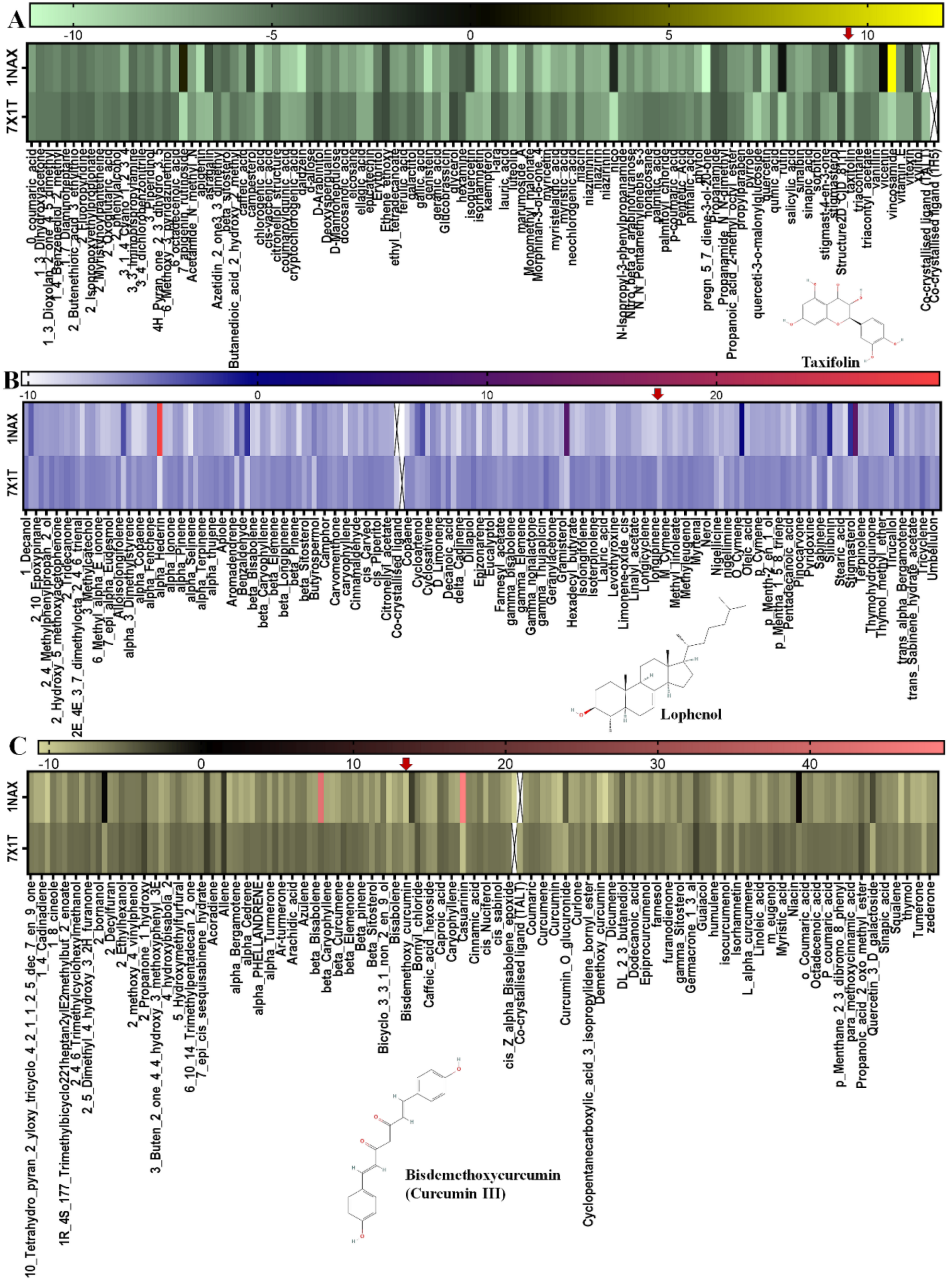
Heatmap representation of the molecular docking scores of phytochemicals identified in selected medicinal plants against the studied target proteins. **(A)***Moringa oleifera*, **(B)***Nigella sativa*, and **(C)***Curcuma longa*.

On 7X1T, the co-crystallized ligand has a binding affinity of -8.67kcal/mol, but lophenol, valoneic acid dilactone, and taxifolin have decreasing binding affinities of -8.07, -7.91, and -7.29 kcal/mol, respectively. **Figure 1** depicts the heatmaps of the screened phytochemicals with an arrow (red) on the potential leads from each plant. The top two (2) lead compounds with lower binding affinities on both receptors for each plant were identified as shown in **Table 1**.

**Table 1:** The binding affinities (docking scores) and the inhibition constants of the selected compounds.

Plants	Code	Phytochemical	7X1T		1NAX	
			Binding affinity	Ki (nM)	Binding affinity	Ki (nM)
***Curcuma longa***	T1	**Valoneic acid dilactone**	-7.91	1590	-10.21	32.75
	T2	Curcumin III	-6.81	10120	-9.66	82.89
***Moringa oleifera***	T3	**Taxifolin**	-7.29	4500	-8.28	854.88
	T4	Luteolin	-6.66	13160	-8.65	455.12
***Nigella sativa***	T5	**Lophenol**	-8.07	1220	-13.62	0.1037
	T6	Stigmastanol	-8.71	415	-12.7	0.4869
**Standards**	T7	CCL (IH5)	–	–	-11.44	4.09
	T8	CCL (Taltirelin)	-8.67	442	–	–
	T9	Levothyroxine	-7.64	2530	-11.60	6.63

CCL denotes Co-Crystallized Ligand, Ki represents the inhibition constant The bold words are phytochemicals with better performance on both receptors.

### Docking validation

**Figure 2:**
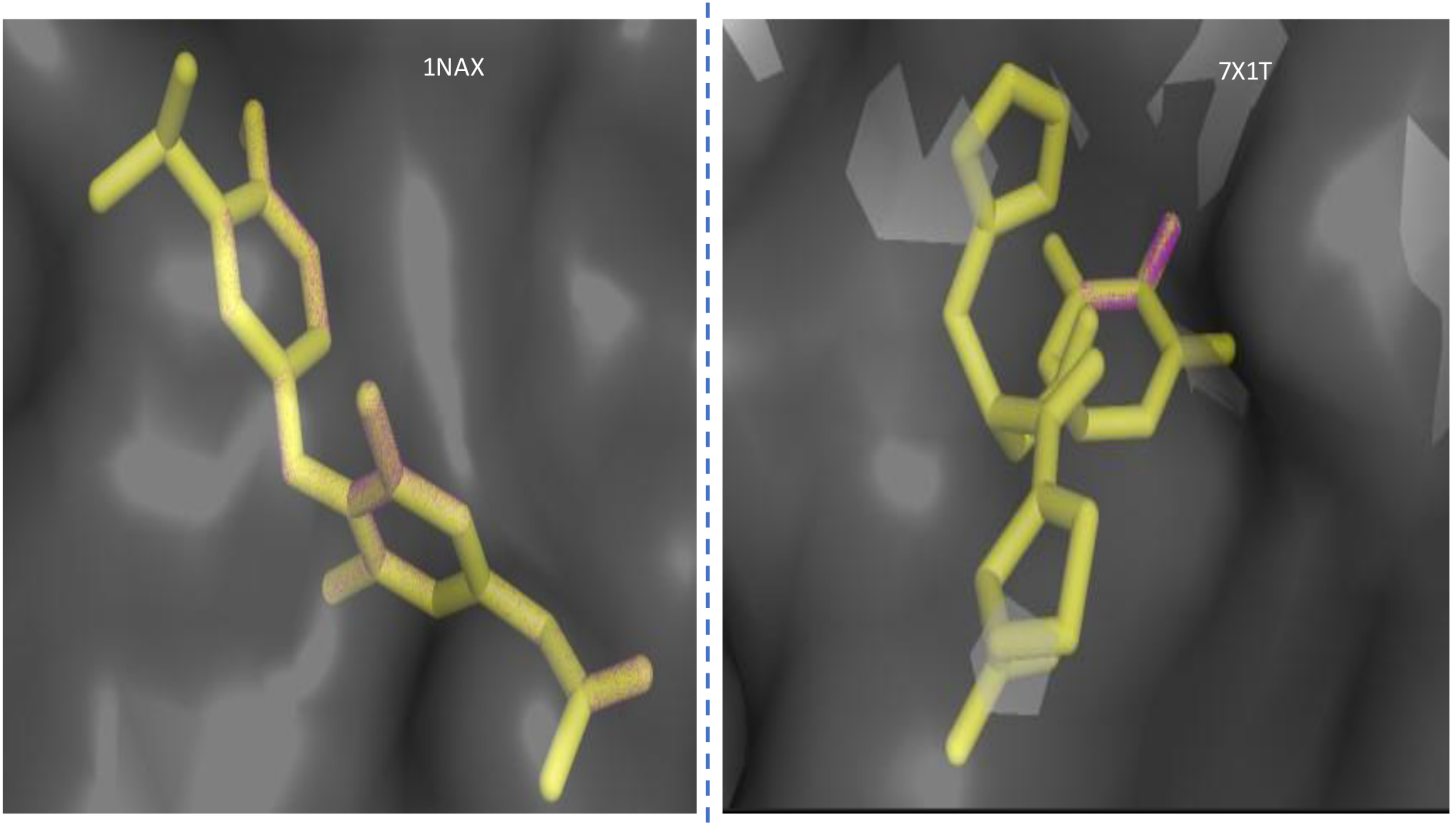
Superimposed structures of the co-crystallised ligands in their co-crystallised (magenta) and re-docked poses (yellow) at the active site of the two receptors 1NAX and 7X1T.

The validation to check the accuracy of the docking procedure was done by re-docking the co-crystallised ligands into the binding sites of the receptor. The low root-mean-square deviation (RMSD) values of 0.000 Å (1NAX) and 0.000Å (7X1T) established the consistency of the grid parameters. Superimposed ligand structures are shown in **Figure 2**.

### Ligand interaction network

**Figure 3:**
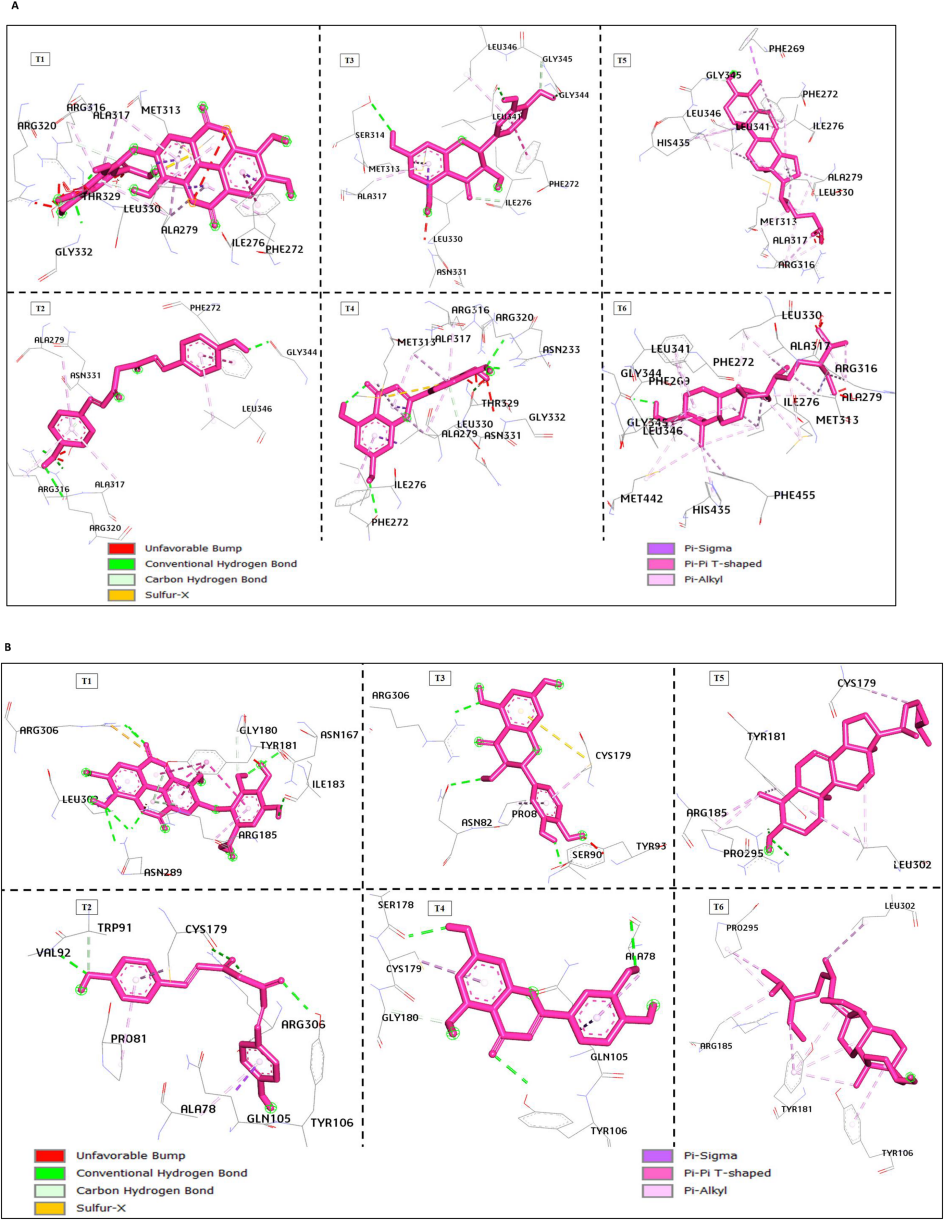
**(A)** The interaction networks between phytochemicals (in magenta) and receptor 1NAX. **(B)** The interaction networks between phytochemicals (in magenta) and receptor 7X1T.

Common key network interactions of the hit compounds, valoneic acid, taxifolin, and lophenol with the amino residues of 1NAX include Arg320, Arg282, Arg316, Leu330, and His435. While hydrogen bonds and hydrophobic interactions are the major interaction networks, some formed water bridges, while others formed salt bridges (**Table 2**; **Figures 3a, b**).

**Table 2:** Ligand interaction types and amino acid residues at 1NAX AND 7X1T.

	Hydrophobic interaction	Water bridge	Hydrogen bond	Salt bridges
1NAX				
**T1**	**Ala279**, Phe272, Ala317, Leu330, Ile276	Arg320	**Arg282**, **Arg 316, Gly332**	–
**T3**	Ala317, Met313, Leu346, Phe272, Leu330	Asn331	Asn82, Ser90, Val92, Tyr93, Gln105, Tyr106, **Arg282**, **Arg306**	–
**T5**	Phe269, Phe272, Thr273, Ile275, Ile276, **Ala279**, Met313, Ala317, Leu330, Leu341, Leu346, **His435**	–	Gly345	–
**T7**	Phe272, Thr273, Phe455	**Arg316, Arg320**	**Asn331, His435**	**Arg282**
**T9**		Arg282, Arg320	Asn233, **Thr329,** Asn331, Gly332, His435	Arg320
7X1T				
**T1**	Leu302, Tyr181	**-**	Asn289, Asn167, Ile183, Tyr181, Arg185	Arg306, Arg185
**T3**	–	–	Asn82, Ser90, Val92, Tyr 93, **Gln105**, **Tyr106**, **Tyr282**, **Arg306**	–
**T5**	Asp85, Ser 178, Tyr181, Arg185, Leu302	–	**Arg185**, **Asn289**	–
**T8**	Leu302	–	**Gln105**, Ser178, Tyr181, Arg185, **Asn289**, Phe296, **Arg306**	
**T9**	Tyr181, Tyr106, Arg306	–	Ala78, **Gln105,** Tyr310	**Arg306**

Amino acid residues obtained from PLIP where the bold ones are key interacting residues.

### In silico ADMET properties and drug likeness

**Figure 4:**
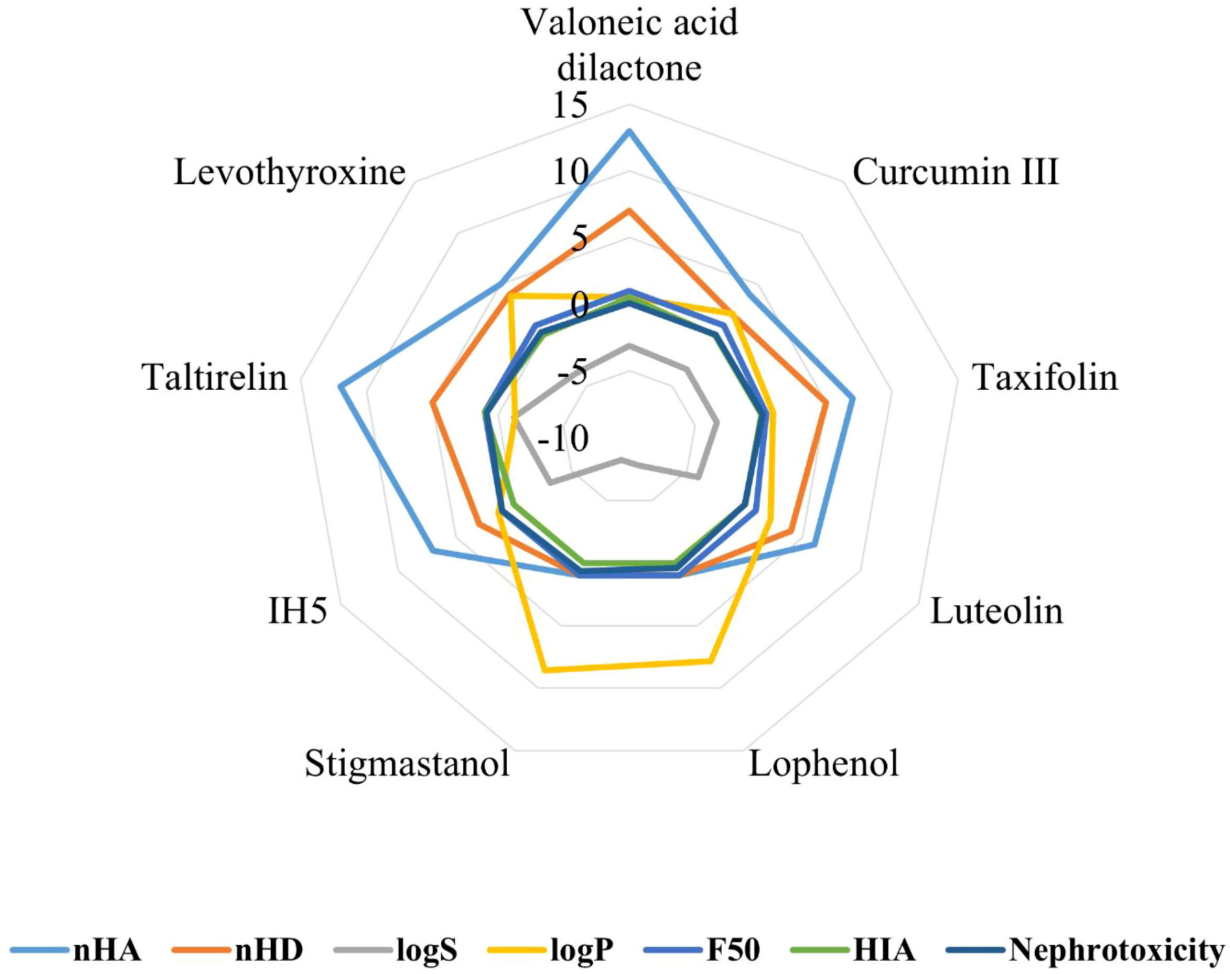
The radar chart of selected physicochemical properties of hits from *M. oleifera*, *N. sativa*, and *C. longa* phytochemicals.

The ADMET properties of hit compounds and standards were evaluated using ADMET Lab 3.0. Valoneic acid dilactone and Taltirelin violated the Lipinski rule of 5 because of the number of hydrogen bond acceptors, which exceeded 10. Details are given in **Table 3** and **Figure 4**.

**Table 3:** The in silico ADMET properties of top leads.

Names	MW	nHA	nHD	logS	logP	Lipinski rule	TPSA	F50	HIA	BBB	Nephro toxicity
Valoneic acid dilactone	470.3	13	7	-3.14	0.55	2	228.3	0.9987	0.4878	No	0.060
Curcumin III	308.3	4	2	-3.34	2.12	0	74.6	0.9987	0.0009	No	0.066
Taxifolin	304.25	7	5	-3.33	0.93	0	127.5	0.4254	0.0660	No	0.126
Luteolin	286.24	6	4	-4.02	2.25	0	111.1	0.9723	0.0146	No	0.001
Lophenol	400.7	1	1	-7.78	7.82	0	20.23	0.9815	1.8E-05	No	0.399
Stigmastanol	416.7	1	1	-8.22	8.59	0	20.23	0.9833	5.3E-05	No	0.656
IH5	389.11	7	3	-3.18	1.31	0	113.4	0.9984	0.0008	No	0.991
Taltirelin	405.18	12	5	-1.23	-1.31	1	170.6	0.9988	0.9787	No	0.857
Levothyroxine	776.69	5	4	-3.72	3.86	0	92.78	0.9741	0.0428	No	0.324

MW, Molecular Weight; nHA, number of Hydrogen acceptor; nHD, number of hydrogen donor; logS, log of Solubility; logP, log of Partition coefficient; TPSA, Topological Polar Surface Area; F50, 50% Bioavailability; HIA: BBB, Blood Brain Barrier.

### Molecular dynamics simulation analysis

The best ligand from each plant was selected based on the low binding affinity, key molecular interactions, and ADMET properties. The two major targets that were of concern in hypothyroidism conditions are Thyroid receptor beta 1 and Thyrotropin-Releasing Hormone receptor.

**Figure 5:**
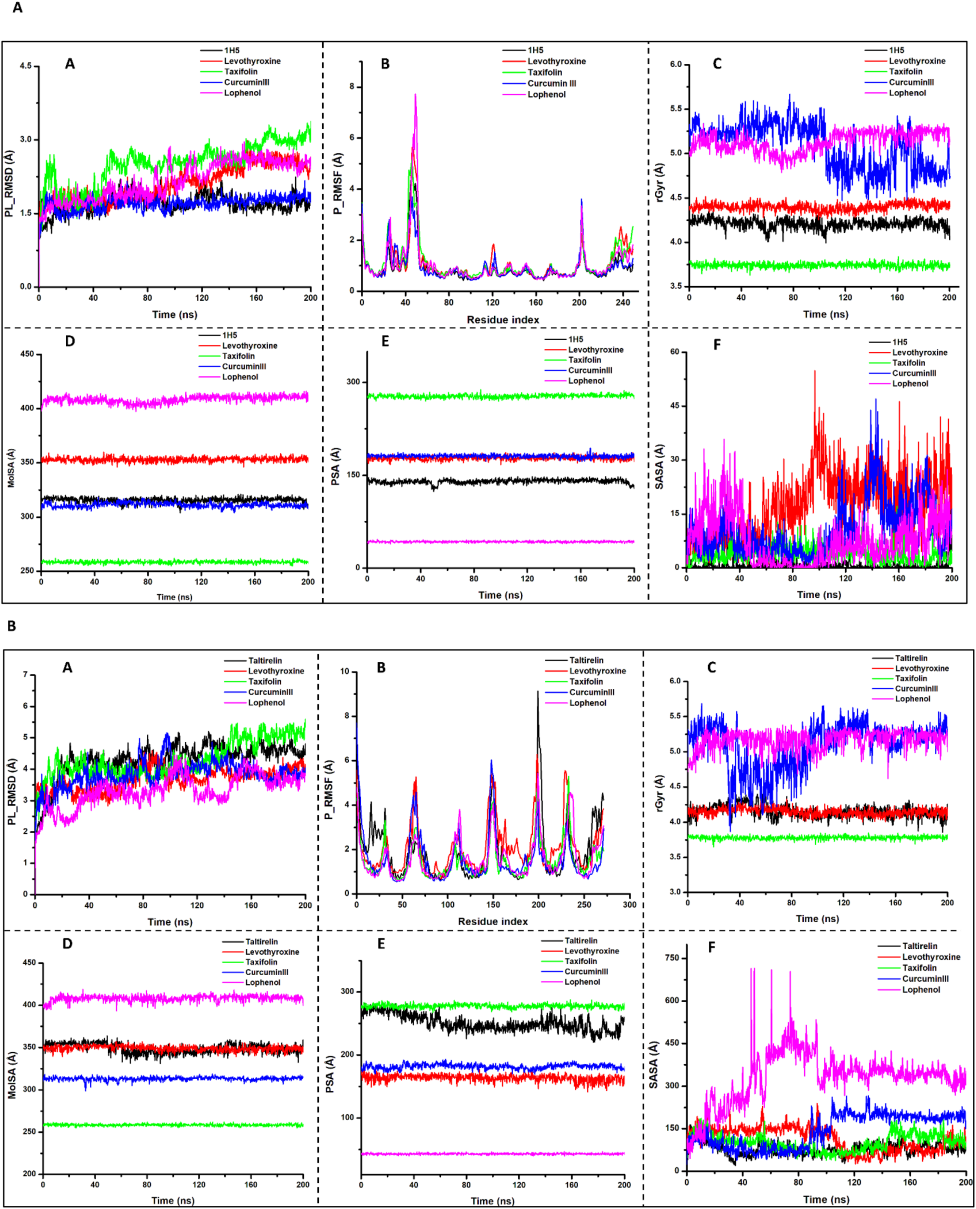
**(a)** The MD simulation results for hit compounds and co-crystallised ligands on the receptor 1NAX. Note: Plots **(A)** Protein-Ligand Root Mean Square Deviation (PL_RMSD); **(B)** Protein Root Mean Square Fluctuation (P_RMSF); **(C)** Radius of Gyration (rGyr); **(D)** Molecular Surface Area (MolSA); **(E)** Polar Surface Area (PSA); **(F)** Solvent-Accessibility Surface Area (SASA). **(b)** Shows MD simulation findings for hit compounds on the receptor 7X1T Note: Plots **(A)** Protein-Ligand Root Mean Square Deviation (PL_RMSD); **(B)** Protein Root Mean Square Fluctuation (P_RMSF); **(C)** Radius of Gyration (rGyr); **(D)** Molecular Surface Area (MolSA); **(E)** Polar Surface Area (PSA); **(F)** Solvent-Accessibility Surface Area (SASA).

For the simulation period of 200ns, the protein and ligand properties, including protein-ligand root mean square deviation (PL_RMSD), protein root mean square fluctuation (P_RMSF), radius of gyration (rGyr), molecular surface area (MolSA), polar surface area (PSA), solvent-accessibility surface area (SASA) were analysed with average values calculated and recorded as shown in **Table 4** and the plots were represented in **Figures 5a, b**.

**Table 4:** The simulation results of the complexes of the hit compounds on the receptors.

Receptor	Protein or ligand property	Curcumin III	Taxifolin	Lophenol	Levo-thyroxine	Co-crystallised ligand
1NAX	**PL-RMSD**	1.70 ± 0.005	2.50 ± 0.014	2.17 ± 0.012	2.10 ± 0.012	1.70 ± 0.006
	**P-RMSF**	0.924 ± 0.035	1.09 ± 0.049	1.06 ± 0.058	1.08 ± 0.051	0.92 ± 0.043
	**SASA**	10.3 ± 0.232	3.92 ± 0.073	7.70 ± 0.217	17.4 ± 0.283	0.713 ± 0.110
	**PSA**	180.7 ± 0.079	277.6 ± 0.087	42.73 ± 0.039	177.5 ± 0.101	140.3 ± 0.110
	**rGyr**	5.08 ± 0.008	3.74 ± 0.001	5.13 ± 0.004	4.40 ± 0.001	4.21 ± 0.002
	**MolSA**	311 ± 0.0702	258 ± 0.040	408 ± 0.104	353 ± 0.066	316 ± 0.075
7X1T	**PL-RMSD**	3.82 ± 0.014	4.29 ± 0.019	3.31 ± 0.017	3.67 ± 0.012	4.29 ± 0.018
	**P-RMSF**	1.58 ± 0.067	1.52 ± 0.051	1.59 ± 0.056	2.09 ± 0.070	1.87 ± 0.079
	**SASA**	143 ± 1.84	102 ± 0.960	329 ± 2.90	114 ± 1.30	79.9 ± 0.733
	**PSA**	181 ± 0.125	278 ± 0.099	43.0 ± 0.039	164 ± 0.166	251 ± 0.406
	**rGyr**	5.10 ± 0.010	3.78 ± 0.001	5.17 ± 0.004	4.15± 0.002	4.16 ± 0.003
	**MolSA**	313 ± 0.066	259 ± 0.039	408 ± 0.115	349 ± 0.097	349 ± 0.181

Protein-ligand root mean square deviation (PL_RMSD), protein root mean square fluctuation (P_RMSF), radius of gyration (rGyr), molecular surface area (MolSA), polar surface area (PSA), solvent-accessibility surface area (SASA). All values in this table are measured in Angstrom (Å).

Protein and ligand properties of hit compounds across the 1NAX receptors are shown in **Table 5** and **Figure 4a**. An RMSD value measures the system stability throughout the simulation time. The co-crystallized ligand (IH5) exhibited the lowest RMSD value, closely similar to curcumin III RMSD value of 1.70 ± 0.006Å and 1.70 ± 0.005 Å, respectively. The lead compounds, taxifolin and lophenol, have close values with the standard drug, levothyroxine (2.50 ± 0.014 Å, 2.17 ± 0.012 Å, and 2.10 ± 0.012 Å, respectively). These values, though higher than that of the co-crystallized ligand, still fall within the acceptable RMSD fluctuation range of 1 and 3Å. While the leads, taxifolin and lophenol, displayed fluctuations within the first 40ns, curcumin III was quite stable during the simulation time.

**Table 5:** The MM/GBSA binding free energy estimation of the protein–ligand complex using the MD trajectories of the hit compounds.

PDB	Protein or ligand property	Curcumin III	Taxifolin	Lophenol	Co-crystallised ligand
1NAX	**ΔE_vdW_**	-52 ± 0.29	-44 ± 0.23	-69 ± 0.34	-52 ± 0.22
	**ΔE_elec_**	-26 ± 0.51	-27 ± 0.39	-5.3 ± 0.02	-10 ± 1.5
	**ΔE_H-bond_**	-1.5 ± 0.05	-1.6 ± 0.03	-0.31 ± 0.03	-3.2 ± 0.05
	**ΔE_covalent_**	2.6 ± 0.12	1.9 ± 0.09	1.4 ± 0.09	3.6 ± 0.11
	**ΔG_solv-GB_**	24 ± 0.19	23 ± 0.20	19 ± 0.18	17 ± 1.30
	**ΔG_bind_**	-80 ± 0.69	-67 ± 0.40	-96 ± 0.62	-75 ± 0.45
7X1T	**ΔE_vdW_**	-40 ± 0.30	-27 ± 0.34	72 ± 103	4.29 ± 0.018
	**ΔE_elec_**	-14 ± 0.64	-26 ± 0.47	-2.4 ± 0.38	3.1 ± 0.12
	**ΔE_H-bond_**	-0.6 ± 0.04	-3.1 ± 0.05	-0.20 ± 0.03	-3.0 ± 0.05
	**ΔE_covalent_**	2 ± 0.21	1.6 ± 0.13	511 ± 509	3.1 ± 0.12
	**ΔG_solv-GB_**	17 ± 0.45	28 ± 0.22	11 ± 0.73	35 ± 0.52
	**ΔG_bind_**	-55 ± 0.56	-35 ± 0.64	574 ± 612	-52 ± 0.32

Values are presented as mean ± standard error of mean (SEM) measured in kcal/mol.

The stability of individual amino acid residues at the active sites of a protein is measured by RMSF analysis (60). The average RMSF value from the lowest, 0.92 ± 0.043 Å of IH5, similar to that of curcumin III, 0.924 ± 0.035 Å to the highest value exhibited by taxifolin (1.09 ± 0.049 Å), all indicated minimal fluctu ations in the active binding site region, hence revealing stable ligand-binding. In **Figure 4a** (B), unstable fluctuations of the amino acid residues were observed in the different ligand systems within the regions 40-55, where lophenol showed the highest fluctuation, while curcumin III has the lowest.

The plots of radius of gyration (rGyr), which analyse protein stability and compactness, revealed that the compounds were stable. The average value of lophenol is the highest (5.13 ± 0.004 Å) and taxifolin exhibited the lowest rGyr (3.74 ± 0.001 Å). For Curcumin III and Lophenol, their Gyr values are quite higher than the values of the standard drug and the co-crystallized ligand (5.08 ± 0.008 Å, 5.13 ± 0.004 Å, 4.40 ± 0.001 Å, 4.21 ± 0.002 Å, respectively).

On the 7X1T, as shown in **Figure 4b**, PL-RMSD values of co-crystallized ligand (taltirelin) and taxifolin are quite similar, and the highest value recorded (4.29 Å), while lophenol, levothyroxine, and curcumin III were 3.31 ± 0.017 Å, 3.67 ± 0.012 Å, and 3.82 ± 0.014 Å, respectively. These values exceeded 3.0 Å, showing that the ligands are not very stable on this receptor. The RMSF values of curcumin III, taxifolin, and lophenol are lower (1.58 ± 0.067 Å, 1.52 ± 0.051 Å, 1.59 ± 0.056 Å) than that of the co-crystallized ligand (1.87 ± 0.079 Å). These values are much lower than levothyroxine (2.09 ± 0.070 Å). The plots of rGyr showed minimal fluctuations; hence, the compounds were stable in the binding pocket.

The values of PSA and MolSA are relatively similar for each of the ligands, but the values of SASA on the receptors. For instance, curcumin III’s PSA value on 1NAX and 7X1T are 180.7 ± 0.079 Å and 181 ± 0.125 Å, respectively, its MolSA is 311 ± 0.0702 Å and 313 ± 0.066 Å, respectively, but its SASA values are 10.3 ± 0.232 Å and 143 ± 1.84 Å, respectively.

The MM/GBSA binding free energy calculations of the complex, as shown in **Table 5**, provides a broad overview of the energy breakdown including van der Waals interaction energy (ΔE_vdW_) as well as binding free energy (ΔG_bind_) for Curcumin III, Taxifolin, and Lophenol compared to the co-crystallized compounds for the two different protein targets 1NAX and 7X1T. The positive ΔE_vdW_ values (72 ± 103 kcal/mol) and exceptionally high positive ΔG_bind_ values (574 ± 612 kcal/mol) for Lophenol showed the unfavourable and unstable binding to 7X1T. Curcumin III showed optimal and stable binding with the two targets, having ΔE_vdW_ -52 ± 0.29 kcal/mol, ΔG_bind_ -80 ± 0.69 kcal/mol, on 1NAX and ΔE_vdW_ -40 ± 0.30, kcal/mol, ΔG_bind_ -55 ± 0.56 kcal/mol on 7X1T. Taxifolin has its ΔE_vdW_ -44 ± 0.23 kcal/mol, ΔG_bind_ -67± 0.40 kcal/mol and 1NAX, and ΔE_vdW_ -27 ± 0.34 kcal/mol, ΔG_bind_ -35 ± 0.64 kcal/mol on 7X1T.

The phytochemicals of *Curcuma longa*, *Moringa oleifera*, and *Nigella sativa* were screened as potential agonists of the TRβ1 (1NAX) and TRHR (7X1T) receptors where curcumin III was found to be the most promising as it formed stable interactions on both receptors, while Taxifolin showed moderate binding properties in the computational study discussed extensively.

## Discussion

In silico analysis has provided great advancement in the discovery of drugs and the study of diseases at the molecular level using computational skills. It performs a key function in the comprehension of protein–ligand interaction and in the prediction of drug-likeness properties of any compound, which is crucial for bench-to-bedside drug development (61). Hypothyroidism treatment with conventional drugs, namely, levothyroxine and liothyronine, is accompanied by side effects. Therefore, the discovery of novel anti-hypothyroidism drugs has become crucial, birthing the invaluable need for evaluation of small molecules as a substitute for hypothyroidism treatment. One of the novel options in the last decade for the enhancement and management of several disease conditions has been the use of bioactive plant compounds, so that there can be minimal side effects of drugs on humans (62). Studies on the treatment of hypothyroidism using medicinal plants are limited, (41). This present study focuses on investigating the activation potential of the hit bioactive compounds across board from *Moringa oleifera*, *Curcuma longa*, and *Nigella sativa* against the active site of the clinical targets. Thyroid receptor beta 1 and Thyrotropin-Releasing Hormone receptor. Molecular docking, molecular dynamics simulation, ADMET properties, among others, are a few of the methods employed.

Systematically, valoneic acid dilactone, curcumin III, taxifolin, luteolin, lophenol and stigmastanol interacted with basic residues such as Arg282, Asn331, Arg320, His435, and Arg316, Gly332, Thr329, Ala279, similar to known activators of Thyroid receptor beta 1 (TRβ). The co-crystallised ligand of the TRβ 1 N-{3-[(4s, 5r)-2-Amino-5-Fluoro-4-(Fluoromethyl)-5, 6-Dihydro-4h-1, 3-Oxazin-4-Yl]-4-Fluorophenyl}-5-Cyanopyridine-2-Carboxamide formed hydrogen bonds with Asn331 and His435 amino acid at the pocket of the active site (45). This finding agrees with prior scholarly work on the structural analysis of selective agonists targeting thyroid hormone receptor β using 3D-QSAR and molecular docking methodologies. It has been established that the specific binding pocket in which the agonists bind contains both polar (Arg282, Ser314, Arg316, Asn331) and nonpolar (Phe269, Phe272, Ile275, Ile276, Ala279, Met310, Ile312, Met313, Ala317, Leu330, Leu346, His435, Phe459) amino acids (63). The presence of a hydrophobic interaction observed between the co-crystallised ligand and the amino residues at the pocket site of the TRβ 1 (N-{3-[(4s, 5r)-2-Amino-5-Fluoro-4-(Fluoromethyl)-5, 6-Dihydro-4h-1, 3-Oxazin-4-Yl]-4-Fluorophenyl}-5-Cyanopyridine-2-Carboxamide) was absent in the interaction occurring between the standard drug (levothyroxine) and the binding site residues. The drug's pharmacokinetics are profoundly influenced by hydrophobic interactions, which are indispensable for the drug's engagement with its biological targets. The affinity and specificity of the drug-receptor interaction depend on hydrophobic interactions, which are the primary features stabilising the drug within this binding site (64). Hence, this elucidates the underlying reason for the adverse effects of Levothyroxine.

For taxifolin, the three hydrogen bonds to Arg282, Arg316 and Gly332, the water bridge formation with Asn331, and the hydrophobic interaction with Phe272 collectively improved binding affinity by making best use of corresponding electrostatic and hydrophobic contacts in the TRβ pocket. Asn331, through a structured hydrogen bonding network, facilitates isoform-specific binding, which is a critical switch for β-selective thyromimetics. The promising stabilisation of the active conformation, facilitated by hydrogen bond and hydrophobic interaction, etc., enables the agonist to activate the receptor and initiate drug action in TRβ, thereby aiding in TRβ-targeted drug development for hypothyroidism (64). This binding mode implies that taxifolin has the potential to be developed to ameliorate hypothyroidism symptoms largely through TRβ activation, contingent upon *in vitro* and *in vivo* data supporting that this docking pattern relates to functional β-selective agonism.

Curcumin III also has two well-positioned oxygen atoms in an aromatic ring that can form a bidentate hydrogen bonding network with amino acids,  Arg316 and Arg320. It participates in a π–π interaction with Phe272. Together, these interactions increase ligand affinity and drive a receptor conformation that is agonistic only in TRβ, as long as the pocket geometry in TRβ allows this arrangement to be accommodated. This structural correspondence provides the most elementary mechanism for selective activation of TRβ and provides clear directions toward its rational optimisation and experimental verification.

Also, the basic residues akin to activating thyrotropin-releasing hormone receptors (TRHR) include Gln105, Tyr106, Tyr282, Asn298, Arg306, Arg185, Tyr181, and Tyr310 (46), whereas the co-crystallized ligand- taltirelin forms a hydrogen bond with Gln105, Asn289, Arg306, Tyr181, and Arg185 and hydrophobic interaction with Leu302. A comparable interaction was also noted for valoneic acid dilactone, curcumin III, taxifolin, luteolin, lophenol, and stigmastanol within the active site. The likelihood of Curcumin III to exhibit selective TRHR stimulation is due to its β-diketone and phenolic oxygens forming a firm, multi-point hydrogen bond network with Arg316 and Tyr106, while its aromatic rings take part in π-π stacking with Phe272. These amino acids arrange the ligand optimally to effectively stabilise the active conformation of TRHR, resulting in selective activation of the receptor. This categorises Curcumin III as a promising compound for developing therapeutic agents aimed at TRHR within the context of hypothyroidism because activation of TRHR promotes physiological TSH release and reinstates normal thyroid hormone synthesis. This implies that the potency of the interaction increases due to the numerous hydrogen bond interactions, which facilitate unique hydrogen-bonded interactions when compared to levothyroxine.

Taxifolin possesses the capability to selectively activate TRHR owing to the presence of phenolic hydroxyl and carbonyl groups, allowing a multi-point H-bond network with Gln105, Tyr106, Tyr282, and Arg306. The above residues offer a unique polar-aromatic microenvironment within the receptor’s binding pocket. The contact and interaction with Gln105 help to stabilise the molecule; a dual-pronged contact by the Tyr106-Tyr282 module secures, in an effective way, the flavanonol ring system through H- and π-interactions, and a solid directional H-bond by Arg306 helps to anchor the receptor firmly into its active conformation. The described topology together culminates in TRHR selectivity, thus indicating that Taxifolin or its derivatives may represent a new class of upstream therapeutics for managing hypothyroidism by triggering normal TSH secretion through targeted activation of TRHR.

From the ADMET results, two of our lead compounds, Curcumin III and taxifolin from *Curcuma longa* and *Moringa oleifera*, respectively, gave good results in relation to how effectively they would be absorbed, distributed, metabolized, and excreted. From the literature, the LogS result above (-5) indicates an overall efficacy of the compounds. From the results, Valoneic acid dilactone (-3.14), Curcumin III (-3.34), taxifolin (-3.35), and luteolin (-4.02) are within the accepted range, while lophenol (-7.78) and stigmastanol (-8.22) could not suffice. Their logP results also show that valoneic acid dilactone, curcumin III, taxifolin, and luteolin have their values less than 3.0. This shows a better result than the standard drug and the co-crystallized ligand (Taltirelin), whose liposolubility is a little higher than 3.0 and less than -1, respectively. This result suggests that they can penetrate the lipid membrane, and since any compound that will be effective for treating hypothyroidism must be able to penetrate the lipid membrane (65, 66). The selected compounds in this study give a better result. The high liposolubility of lophenol and stigmastanol makes it difficult to penetrate the lipid membrane. TPSA range for an effective hypothyroidism drug is between 60-140 Å (67). Fortunately, Curcumin III has 74.6 Å^2^, taxifolin 127.5 Å^2^, luteolin 111.1Å^2^, levothyroxine 92.78 Å^2,^ and co-crystallized compound (1H5) 113.4Å^2^ compared to the co-crystallized compound (taltirelin) with 170.6 Å. All the selected compounds are more likely to have good oral bioavailability, having followed the Lipinski rule of five, excluding valoneic acid dilactone and taltirelin (68). The ADMET result, however, confirms that curcumin and taxifolin will give better results compared to levothyroxine.

Additionally, the stability of protein-activator complexes was estimated by performing Molecular Dynamics (MD) simulations. The principal tool in computational chemistry to acquire a thorough report on the fluctuations and conformational shifts of proteins and ligands in a time-bound approach is MD simulation (69). The results of the molecular dynamic simulation, particularly, the RMSD, RMSF, protein-ligand contact, and ligand properties show that the choice compounds are quite stable on both receptors as their RMSD values are within the accepted range of 2-3Å, Curcumin III (1.70 ± 0.005), taxifolin (2.50 ± 0.014 Å), and lophenol (2.17 ± 0.012Å) (70) The P_RMSF results (< 2 Å) are indicative of a stabilized protein. The stabilised binding mode implies that curcumin (0.924 ± 0.035 Å), taxifolin (1.09 ± 0.049 Å), and lophenol (1.06 ± 0.058 Å) provide strong interaction with TRβ 1 and TRHR, thereby improving its activator potential. The low SASA values, high PSA values, and rGyr results of the curcumin and taxifolin on the two receptors suggest the need for optimisation of the compound to enhance its bioavailability.

The MMGBSA results reveal that on the 1NAX protein, all three ligands produce positive van der Waals properties, with negative ΔE_vdW_ values. It is appropriate to note that van der Waals activity in Lophenol is the highest (-69 ± 0.34 kcal/mol) compared with Curcumin III (-52 ± 0.29 kcal/mol), Taxifolin (-44 ± 0.23 kcal/mol), and the co-crystallized ligand (-52 ± 0.22 kcal/mol). This trend is evident in the analysis of binding free energy, too. Lophenol has the best ΔG_bind_ (−96 ± 0.62 kcal/mol), which indicates that Lophenol has the best binding ability for the 1NAX target molecule. Curcumin III has a ΔG_bind_ of (−80 ± 0.69 kcal/mol), which is better when compared to the co-crystallized molecule with a ΔG_bind_ of (−75 ± 0.45 kcal/mol). However, compared to the co-crystallized molecule, Taxifolin has a relatively weaker binding free energy. The binding ability of Curcumin III shows a more stable binding when compared to the binding ability of the co-crystallized ligand (−52 ± 0.32 kcal/mol).

It is significant to note that Lophenol has profoundly unfavourable and unstable binding to 7X1T, as made clear by its positive ΔE_vdW_ values (72 ± 103 kcal/mol) and its exceptionally high positive ΔGbind values (574 ± 612 kcal/mol). Additionally, the large error bars reflect a lack of convergence of the interactions that occurred during the simulation due to steric issues or the inability of Lophenol to adopt a stable bindable conformation in the 7X1T bindable site.

Several compounds, including Taxifolin and Curcumin III, exhibited interactions similar to known receptor agonists. 2-D Ligand interactions revealed that hydrogen bonding and hydrophobic interactions show selective activation of TRβ1 and TRHR. ADMET results ranked Curcumin III and Taxifolin higher because of their solubility, membrane permeability, and oral bioavailability over the other phytocompounds and levothyroxine. Molecular dynamics and MMGBSA analyses confirmed the stability of the binding of Curcumin III and Taxifolin, making them leading dual-target compounds.

## Conclusion

The present study highlights the potential of curcumin III from *Curcuma longa* and taxifolin from *Moringa oleifera as* the most effective activators of the selected dual target of hypothyroidism - thyroid receptor beta 1 and thyrotropin-releasing hormone receptors. The dynamics and steadiness of curcumin III and taxifolin complexes have been proven by carrying out molecular dynamics simulations. Besides, the physicochemical and ADMET properties of curcumin III and taxifolin revealed that they have almost all the properties of a drug-like molecule. The MMGBSA results showed that lophenol has exceptional binding affinity for 1NAX, but it does not bind favorably with 7X1T, which clearly signifies selectiveness. This disqualifies it from being a potential dual target, which this study aims at. Curcumin III shows optimal and stable binding with all the targets and performs better than the ligand used in the co-crystallization process, clearly signifying its potential to be a universal binder. On the other hand, taxifolin shows relatively weaker binding despite its stable nature when compared with the other ligands. The binding stability in taxifolin is an important strength, which clearly implies strong compatibility with the receptor pocket. The relatively low affinity could be explained based on an inadequately addressed hydrophobic/disperse interaction, which could be improved by hydrophobic site modification, rigidity improvement, and site-directed hybridization. By optimizing taxifolin, it has the potential to be developed into a highly effective and selective lead compound for the corresponding molecular targets for hypothyroidism. Nevertheless, the outcomes of this study necessitate *in vitro*, *in vivo* and *ex vivo* endorsement for the verified natural compounds beforehand clinical procedure.

## Data Availability

The original contributions presented in the study are included in the article/supplementary material. Further inquiries can be directed to the corresponding author.
